# Sperm storage in caecilian amphibians

**DOI:** 10.1186/1742-9994-9-12

**Published:** 2012-06-06

**Authors:** Susanne Kuehnel, Alexander Kupfer

**Affiliations:** 1 Institut für Spezielle Zoologie und Evolutionsbiologie mit Phyletischem Museum, Friedrich-Schiller-Universität Jena, Erbertstraße 1, 07743, Jena, Germany; 2 Institut für Biochemie und Biologie, Zoologie, Universität Potsdam, Karl-Liebknechtstraße 24-25, 14476, Potsdam, Germany

**Keywords:** Reproduction, Sperm storage, Amphibians, Caecilians

## Abstract

**Background:**

Female sperm storage has evolved independently multiple times among vertebrates to control reproduction in response to the environment. In internally fertilising amphibians, female salamanders store sperm in cloacal spermathecae, whereas among anurans sperm storage in oviducts is known only in tailed frogs. Facilitated through extensive field sampling following historical observations we tested for sperm storing structures in the female urogenital tract of fossorial, tropical caecilian amphibians.

**Findings:**

In the oviparous *Ichthyophis* cf. *kohtaoensis*, aggregated sperm were present in a distinct region of the posterior oviduct but not in the cloaca in six out of seven vitellogenic females prior to oviposition. Spermatozoa were found most abundantly between the mucosal folds. In relation to the reproductive status decreased amounts of sperm were present in gravid females compared to pre-ovulatory females. Sperm were absent in females past oviposition.

**Conclusions:**

Our findings indicate short-term oviductal sperm storage in the oviparous *Ichthyophis* cf. *kohtaoensis*. We assume that in female caecilians exhibiting high levels of parental investment sperm storage has evolved in order to optimally coordinate reproductive events and to increase fitness.

## Background

Animal reproductive strategies include variable modes of sperm transfer, fertilization, and type of offspring development. In particular female sperm storage, where male spermatozoa remain in the reproductive tract after mating until used for fertilization, has evolved independently and repeatedly in metazoans as a mechanism to temporarily decouple insemination from fertilization [1,2]. In vertebrates, female sperm storage in dedicated structures occurs in all major lineages with durations ranging from a few hours or days in most mammals (not including bats) to long-term storage up to months in sharks, turtles, birds and also reptiles with a reported maximum of seven years [2]. Among modern amphibians many female salamanders can store sperm in unique cloacal spermathecae [3] and internal fertilising anurans such as tailed frogs (*Ascaphus* ssp.) have sperm storage in the oviducts [4]. This raises the question whether female sperm storage has also evolved in the third group of extant amphibians, the limbless caecilians [5,6].

**Figure 1:**
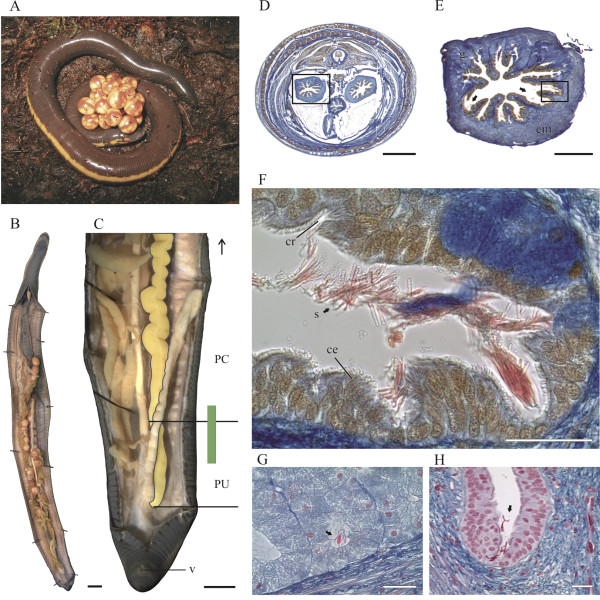
**Sperm storage in*Ichthyophis*cf.*kohtaoensis*. A:** Female guarding a clutch of eggs. **B:** Viscera of a preovulatory female. **C:** Posterior body with oviduct outlined, horizontal lines indicate border between different oviductal parts. Green bar indicates region of sperm storage. **D–G:** Azan stained cross sections of preovulatory females: One female indicating the transition between PC and PU of the left oviduct (D, E) with regressing glands and sperm masses in detail (F) between the mucosal folds and epithelial crypts. G. Spermatozoa stored in tubules of simple tubular glands of the PC. **H:** Azan stained cross section showing the PU of a gravid female with fewer sperm. Scale bars: A, B 5 mm. D 2 mm. E 500 μm. F 100 μm. G, H 50 μm. ce = ciliated epithelium, cm = circular musculature, cr = epithelial crypt, g = glands, PC = pars convoluta, PU = pars uterina, s = sperm, v = vent. Arrows indicate sperm masses.

Caecilians perform internal fertilization with the aid of an intromittent organ [7,8] and show various extraordinary reproductive strategies including maternal dermatotrophy and intrauterine feeding [9,10]. We investigated the potential for sperm storage in a species with high parental investment (*Ichthyophis* cf. *kohtaoensis*, see Figure 1A). Apparently reproductive success is strongly dependent on environmental conditions such as temperature and humidity. Under fluctuating conditions sperm storage in specialized compartments in either the cloaca or the oviduct (or both) might be highly adaptive. Yet, except for an historical observation of live sperm in the female oviduct more than a century ago [11] nothing is known. We investigated this open question in field collected female *Ichthyophis* cf. *kohtaoensis* around the breeding season and found evidence of sperm presence and storage in caecilian amphibians.

## Findings

Paired oviducts are located lateral to the kidneys. Extending anteriorly to the heart they terminate in small ostia, which receive the eggs and they join the cloaca posteriorly (Figure 1B). Of seven female specimens collected prior to egg-laying four, contained mature, yolky ovarian follicles (MD = 6.4 mm) and another three already had fertilized ova located in the convoluted central part (pars convoluta, PC) of the oviduct (see Table 1). Stained slightly red to pink with Heidenhain’s Azan, tripartite spermatozoa with a characteristic flattened head (about 11 μm), a mid-piece, and a single flagellum were found within the posterior oviduct in six of seven vitellogenic females (see Figure 1F–G). To elucidate this region we briefly describe the respective parts of the oviduct. Following the relatively short (1/5) anterior part (pars recta), the substantially convoluted pars convoluta (PC) constitutes more than half the oviduct and comprises two gross morphological and histological different portions. At the level of the posterior end of the ovaries, the posterior part (about ¼) of the PC is well demarcated in reproductively active females by its diameter (about ~2,5 mm) and thick wall that is composed of radially arranged, simple branched tubular glands made of voluminous secretory cells (AB-, intensively PAS+) with well stained, basal nuclei and plasma containing neutral mucopolysaccharids and granules. They are separated into several compartments by septs of connective tissue and orifice into the oviductal lumen. Short prolonging mucosal folds lined with columnar ciliated epithelium that forms crypts, further narrow the lumen. Glands regress at about the level of the posterior end of the kidneys (Figure 1D, E) The remaining straight posterior-most portion resembles the uterine part (PU) of the oviduct, characterised by a thick layer of smooth circular musculature and radially projecting, longitudinal mucosal folds. Those are lined with multi-rowed columnar ciliated epithelium, which is interspersed with non-ciliated secretory cells (PAS+) and frequently divided by cleft like crypts regressing further posteriorly. Oviductal sperm were restricted to a region between the middle of this posterior aglandular PU up to the transition into the posterior part of the glandular convoluted PC (Figure 1C). Spermatozoa were neither found further anteriorly nor in the cloaca and were absent in females collected after oviposition (Table 1).

**Table 1:** **List of female*Ichthyophis*cf.*kohtaoensis*examined and details of their reproductive status**

ID	TL (mm)	collected	season	oviductal sperm	vitellogenic eggs	gravid	clutch
SMNS 11733	398	23.01.2000	dry	–	+	–	–
AK109	349	22.05.2001	rainy	++	+	–	–
AK01673	302	22.05.2001	rainy	++	+	–	–
AK01674	304	22.05.2001	rainy	++	+	–	–
AK01675	350	14.06.2001	rainy	+/–	–	+	–
AK150	336	15.06.2001	rainy	+	–	+	–
AK01364	399	22.06.2001	rainy	+/–	–	+	–
AK149	370	17.06.2001	rainy	–	–	–	+
AK01676	335	07.06.2001	rainy	–	–	–	+
AK01362	330	25.07.1995	rainy	–	–	–	+
AK01363	326	25.07.1995	rainy	–	–	–	+

AK = Alexander Kupfer, Museum abbreviation: Staatliches Museum für Naturkunde Stuttgart, Germany (SMNS).

The amount and localization of sperm varies greatly between gravid and pre-ovulatory females. In the latter, large and medium amounts of free spermatozoa were present in the oviductal lumen of the anterior PU, but were found most abundantly in between the mucosal folds and their epithelial crypts. Spermatozoa were aggregated in bundles and similarly orientated with heads pointing to the ciliated epithelium (Figures 1D–F). Further anteriorly, additional sperm were found within the distal parts of the simple branched tubular glands at the transition into, and within the posterior PC. Again, spermatozoa were similarly orientated (Figure 1G), more or less closely packed and associated with transparent fluid (PAS+) indicating secretions of the glands.

Three gravid females contained sperm restricted to the aglandular PU, however in decreased numbers, compared to the pre-ovulatory condition (Figure 1H). Spermatozoa were rarely detected in the oviductal lumen. Sperm were located between the most distal ends of the longitudinal folds and respective epithelial crypts, sometimes enclosed by the epithelium and often occurring more individually.

## Discussion

Given that oviductal sperm were detected in all females that were collected prior to oviposition, we can refute cloacal sperm storage in *Ichthyophis* cf. *kohtaoensis*, which is in accordance with the report that no cloacal tubules are available for sperm storage [12]. We present evidence that cloacal storage in dedicated spermathecae widespread among the Salamandroidea [3,5], might be lacking in caecilians.

Following the anecdotal observation of living sperm in the posterior oviducts of a single female caecilian, *I. glutinosus*[13], we are able to fully verify oviductal sperm in another oviparous species in this genus. The presence of spermatozoa (1) in a distinct region (2) prior to and after ovulation, and (3) with a consistent orientation is the first evidence of sperm storage in caecilians. Sperm were found in two subsequent but morphologically different sites in the posterior oviduct. The distal glandular tubules of the posterior PC could possibly resemble distinct sperm storage tubules (SSTs) similar to seminal receptacles in the oviducts of birds or squamates. In snakes and lizards, SSTs are compound tubular or alveolar glands located anteriorly between the uterus and infundibulum [14-16]. Other squamates possess additional, vaginal SSTs and iguanid lizards such as the green anoles, fully replaced anterior receptacles [17,18]. But the prominent glands of the posterior convoluted oviduct seen in female caecilians differ from SSTs. Evidently sperm were particularly frequent in the elongated uterine part between the mucosal folds and epithelial crypts. Oviductal sperm storage in the absence of glands, but within deep, narrow furrows and “crypts” of longitudinally folds is also known for several squamates such as red-sided garter snakes, *Thamnophis sirtalis parietalis*[19] and ground skinks, *Scincella lateralis*[20].

However, the general morphology of the posterior oviduct and anatomical site of sperm presence in *Ichthyophis* cf. *kohtaoensis* more closely resembles the situation found in *Ascaphus truei*, so far the only other amphibian known to have oviductal sperm storage. In *A. truei* sperm is stored in simple tubular glands equipped with ciliated and secretory cells of the elongated ovisac, the posterior-most part of the oviduct [4]. No generalized information about the exact physiological mechanisms of SSTs is available so far [2]. Such receptacles of both squamates and Ascaphus truei are basically continuations of the epithelium and especially in the latter they were identified as sites for sperm residence rather than physiologically dedicated for storage [5,20]. Such a functional significance of the PU and tubules of the posterior PC serving as temporal sperm receptacles after copulation, we would suggest for caecilians as well. Because sperm were not found more anterior in the PC and are totally absent after ovulation, an additional physiological border eventually represented by gland secretions might exist preventing the passage of spermatozoa along the entire oviduct just until ovulation. But the mechanisms of sperm release for fertilisation remain unknown.

*Ichthyophis* cf. *kohtaoensis* likely reproduce annually or biannually [21]. Reproduction is highly correlated with the rainy season: in late May, shortly after the onset of the monsoon, males and females were present on the breeding site. Thus, females carrying large quantities of oviductal sperm likely would have copulated recently, but because ovulation was not yet initiated, spermatozoa must remain viable to enable fertilization. Because gravid females carrying fertilized eggs and fewer sperm have been collected c. three weeks later we assume sperm storage for at least a few weeks, which also confirms previous field observations of a copulation and subsequent oviposition [22].

Functionally, sperm storage in caecilians would be advantageous if an additional clutch could be fertilized from a single mating. However, evidently this scenario of long-term storage is not applicable because oviductal sperm are absent after oviposition due to the lack of distinct SSTs apart from the main tube, which indicates storage for a single breeding season only. We favour the hypothesis that by further uncoupling reproductive events females might optimize the timing of each event, because one advantage of sperm storage is to reproduce in response to environmental variation [2].

Short-term storage in *Ichthyophis* cf. *kohtaoensis* might also be evolved in correlation with mating. Because subterranean caecilians have a highly specialised olfactory organ, the tentacle, surface associated chemical signalling might be involved in mate finding but currently no experimental evidence exists for this hypothesis. Although reproductive cycles seem to be synchronous, males and females encounter rates might be sufficiently low to favour the evolution of sperm storage. Female investment in offspring is high including a large yolk supply (see Figure 1A and B), the building of subterranean nests for egg-laying and long-term guarding of the clutch (see also Figure 1A). Post-copulatory sperm storage facilitates maternal control in order to increase reproductive success, fitness [23] and to assure previous investment in eggs. Possible multiple matings and relations of post-copulatory female choice but also sperm competition with respect to the varieties among the male copulatory organ remains to be studied.

## Methods

We examined eleven adult females of *Ichthyophis* cf. *kohtaoensis* collected in north-eastern Thailand (Isan region, Mekong Valley, Khemmarat District, Ubon Ratchathani Province, see [21]) during May-June and January-February (see details in Table 1) and fixed in an aqueous solution of 4% formaldehyde. Specimens were opened through a medioventral incision and the reproductive status of the gonads was assessed under a stereomicroscope. To detect sperm and to analyse the oviductal morphology, conventional histology was applied to serial sections of oviducts of vitellogenic specimens and the most cranial, caudal and middle oviduct of gravid females carrying oviductal eggs. In addition the most posterior part including the cloaca of three females was studied (see [12]). After dehydration samples were embedded in paraffin, serially sectioned (8–12 μm) and stained following standard protocols for Heidenhain’s Azan, Alcian Blue (pH 2,5) and classic PAS reaction using nuclear fast red for counterstaining [24]. We adopt the tripartite oviductal terminology of [25].

## Abbreviations

AB: Alcian Blue; c: circa; cf: (confer, latin) - confer; e.g.: (exempli gratia, latin), for example; MD: mean diameter (in mm); PAS: Periodic Acid Schiff; PC: pars convoluta; PU: pars uterina; SSTs: sperm storage tubules.

## Competing interests

The authors declare that they have no competing interests.

## Authors’ contributions

AK collected samples during field-work. AK and SK designed the research. SK carried out histology and analysis in the laboratory. SK and AK interpreted the results and wrote the manuscript. Both authors read, edited and approved the final version of the manuscript.
